# Early detection of myocardial fibrosis in type II diabetic patients using MR T1-mapping

**DOI:** 10.1186/1532-429X-13-S1-O110

**Published:** 2011-02-02

**Authors:** Helene Thibault, Laura Ernande, Stanislas Rapacchi, Magalie Viallon, Cyrille Bergerot, Franck Thuny, Michel Ovize, Han Wen, Genevieve Derumeaux, Pierre Croisille

**Affiliations:** 1 Louis Pradel Hospital and Inserm U886, Lyon, France; 2 CREATIS-LRMN, Lyon, France; 3 Department of Radiology, University Hospitals of Geneva, Geneva, Switzerland; 4 Assistance Publique - Hôpitaux de Marseille, Marseille, France; 5 National Heart, Lung, and Blood Institute, National Institutes of Health, Bethesda, Maryland, USA; 6 Louis Pradel Hospital and CREATIS-LRMN, Lyon, France

## Introduction

Diabetes mellitus may alter cardiac structure and function independently of underlying coronary artery disease or hypertension. This so called “diabetic cardiomyopathy” is associated with myocardial fibrosis and is characterized by a long and silent phase of progressive left ventricular (LV) remodeling before the occurrence of contractile dysfunction and heart failure symptoms. In the preclinical stage, with normal LV ejection fraction, new echocardiographic techniques reported subtle myocardial dysfunction with a decrease in systolic strain.

Recently, myocardial T1 mapping has been proposed to detect interstitial fibrosis early in the disease course.

## Purpose

to evaluate whether myocardial T1 mapping could detect abnormalities in type 2 diabetic patients with normal standard parameters of LV function and normal LGE imaging.

## Methods

Type 2 asymptomatic diabetic patients with no history of heart disease, a normal LV assessed by conventional echocardiography (normal LV volumes, ejection fraction and wall motion) and normal LGE imaging were compared to matched healthy volunteers.

T1 quantification was performed using a Modified Look-Locker Inversion -recovery (MOLLI) sequence at 1.5T (Siemens), on a short axis of the LV, before, 5 and 15 min after 0.2 mmol/kg gadolinium injection. Imaging protocol included also standard Cine-SSFP imaging, and LGE imaging. Regional strains were assessed using Displacement Encoding with Stimulated Echoes (DENSE) imaging. Left ventricular diastolic function (mitral inflow pattern) was further assessed using echocardiography

## Results

Twenty-four diabetic patients (51±4 years old) and 16 matched volunteers (47±7 years old) were included. Despite normal ejection fraction, global circumferential strain was decreased in diabetic patients compared to volunteers (14.6±0.3 vs. 17.0±0.4 %, respectively, p<0.05).

Mean myocardial T1 relaxation time was significantly shorter in diabetic patients than in volunteers both at 5 (312±5 vs.361±6 milliseconds, respectively, p<0.001) and 15 minutes (405±6 vs. 456±5 milliseconds, respectively, p<0.001) after gadolinium injection.

Echocardiography displayed abnormal diastolic LV filling with an impaired LV relaxation in 55% of patients and in 25% of volunteers. Interestingly, post-contrast myocardial T1 time was shorter in case of impaired relaxation than in case of normal mitral inflow pattern (320±8 vs. 340±16 milliseconds, respectively, p=0.05).

## Conclusions

T1 relaxation time is decreased in asymptomatic type 2 diabetic patients with normal ejection fraction. T1 abnormalities are associated with impaired myocardial circumferential strain and early diastolic dysfunction suggesting that interstitial fibrosis may be implicated in diabetic cardiomyopathy. In the future, T1 quantification may contribute to detect subclinical stage of diabetic cardiomyopathy.

